# Eliglustat prevents acute kidney injury caused by Shiga toxin 2 in lethal and sublethal rat models of hemolytic uremic syndrome

**DOI:** 10.3389/fphar.2026.1736204

**Published:** 2026-02-19

**Authors:** Daiana S. Sánchez, Lilian K. Fischer Sigel, Elsa Zotta, Claudia Silberstein

**Affiliations:** 1 Universidad de Buenos Aires (UBA), Facultad de Ciencias Médicas, Departamento de Ciencias Fisiológicas, Laboratorio de Fisiología Renal, Buenos Aires, Argentina; 2 Universidad de Buenos Aires – Consejo Nacional de Investigaciones Científicas y Tecnológicas (CONICET), Instituto de Fisiología y Biofísica Bernardo Houssay (IFIBIO Houssay), Buenos Aires, Argentina; 3 Universidad de Buenos Aires, Facultad de Farmacia y Bioquímica, Departamento de Ciencias Biológicas, Cátedra de Fisiopatología, Buenos Aires, Argentina; 4 Universidad de Buenos Aires, Facultad de Ciencias Médicas, Departamento de Ciencias Fisiológicas. Laboratorio de Patología y Nefrología Experimental, Buenos Aires, Argentina

**Keywords:** eliglustat, globotriaosylceramide receptor, glucosylceramide synthase inhibitor, hemolytic uremic syndrome, renal tubular injury prevention, Shiga toxin type 2

## Abstract

**Background:**

Shiga toxin-producing *Escherichia coli* is the main cause of post-diarrheal hemolytic uremic syndrome (HUS), which produces acute kidney injury mainly in children. Shiga toxin exerts its action by binding to the glycolipid globotriaosylceramide (Gb3) on the cell surface of target tissues. In humans, the kidneys are the organs most affected by Shiga toxin, where high Gb3 expression in glomerular endothelial and tubular epithelial cells leads to a combined glomerulopathy and tubulopathy that drives acute kidney injury. The aim of the present work was to evaluate the effectiveness of oral treatment with eliglustat (EG), an inhibitor of glucosylceramide synthase, the first step of glycosphingolipid biosynthesis, in preventing the effects of Shiga toxin 2 (Stx2) in lethal and sublethal models of HUS in rats.

**Methods:**

Male juvenile Sprague–Dawley rats (∼100 g body weight (bw)) were intraperitoneally injected with lethal (5 ng/g bw) or sublethal (1 ng/g bw) doses of Stx2, or eluent. Some rats were orally treated with EG (15 mg/d or 25 mg/d) for 0–2 days, until 2 days or 3 days post-injection. Rat survival, renal Gb3 expression, renal function, histopathological observations, and aquaporin 1 (AQP1) and neutrophil gelatinase-associated lipocalin (NGAL) tubular expression were evaluated.

**Results:**

Rats injected with the Stx2 lethal dose showed significant kidney injury with extended tubular necrosis and some glomerular alterations at 3 days post-injection. The Stx2 sublethal dose caused significantly less renal injury extension than the lethal dose, showing only tubular damage. The expression of AQP1 and NGAL corroborated the tubular alteration in Stx2-injected rats. The oral treatment with EG reduced Gb3 renal levels and, therefore, significantly prevented the renal injury caused by lethal and sublethal doses of Stx2 in rats. Moreover, the pretreatment for 2 days with EG prevented the mortality in those rats injected with the Stx2 lethal dose. The EG treatment did not cause renal or systemic alterations. The effectiveness of EG treatment depended on the dose and the pretreatment time.

**Conclusion:**

The oral treatment with EG could be a therapeutic strategy to prevent the action of Stx2 and the development of acute kidney injury in diarrhea-associated HUS patients.

## 1 Introduction

Shiga toxin (Stx)-producing *Escherichia coli* (STEC) is the main cause of post-diarrheal hemolytic uremic syndrome (HUS), characterized by hemolytic anemia, thrombocytopenia, and acute kidney injury (AKI) (Gianantonio et al., 1964; Karmali et al., 1985). In Argentina, post-diarrheal HUS caused by STEC is endemic and is one of the most common etiologies of pediatric AKI, which mostly affects children under 5 years, with a case mortality of less than 4% (Rivas et al., 2014; Balestracci et al., 2021; Rivas et al., 2023). Although Argentina was reported to be the country with the highest incidence of HUS caused by STEC infection, outbreaks were reported worldwide, such as in other countries of Latin America (Torres et al., 2018), Europe, North America, Australia, and Japan (Joseph et al., 2020), representing a global health problem. Approximately 30% of the affected children who survive the acute phase of the disease are at risk of developing chronic renal injury and long-term renal sequels (Repetto, 2005; Cobeñas et al., 2007). HUS may cause end-stage renal disease in the pediatric population, leading to approximately 9% of renal transplants in children and adolescents (National System of Procurement and Transplant Information of the Argentine Republic, 2020). Humans can be infected mostly by eating undercooked beef products, fresh fruits and vegetables, and other products that have been contaminated with STEC (Rivas et al., 2014).

Shiga toxin type 1 and 2 (Stx1 and Stx2, respectively) and/or their subtypes (Stx1a, c, d; Stx2a–g) are the main virulent factors produced by STEC O157:H7 and other related bacterial strains, with Stx2a-producing STEC being the most virulent strain that causes HUS (Rivas et al., 2023; Basu and Tumer, 2015). The toxin is composed of an A subunit non-covalently bound to a pentamer of B subunits. The Stx B pentamer binds to the glycosphingolipid globotriaosylceramide, known as Gb3, located on the plasma membrane of target cells in different tissues (O’Brien and Holmes, 1987; Lingwood, 1996). Subsequently, Stx2 is endocytosed and retrogradely transported to the endoplasmic reticulum, and the A subunit exerts RNA *N*-glycohydrolase activity leading to cell death by inducing protein synthesis inhibition and apoptosis (Endo et al., 1988; Sandvig et al., 1992; Johannes and Romer, 2010; Tesh, 2012).

The Gb3 glycosphingolipid is the functional main receptor described for Stx, producing Stx-mediated HUS (Lingwood, 1996; Okuda et al., 2006; Celi et al., 2022). In humans, the kidneys are the organs most affected by Stx, where high Gb3 expression in glomerular endothelial cells, podocytes, mesangial cells, and tubular epithelial cells leads to a combined glomerulopathy and tubulopathy that drives acute kidney injury (Obrig and Karpman, 2012; Legros et al., 2017; Amaral et al., 2013; Sánchez et al., 2021). Different reports documented Stx-mediated glomerular and tubular damage in the kidneys of both pediatric and adult patients with HUS caused by STEC (Uchida et al., 1999; Chaisri et al., 2001; Porubsky et al., 2014). Previous works, conducted in human renal tubular epithelial cells (HRTECs), demonstrated the direct cytotoxic action of Stx2 on the tubular epithelia (Karpman et al., 1998; Creydt et al., 2006; Silberstein et al., 2008; Márquez et al., 2014). Studies in rats (Sugatani et al., 2002; Zotta et al., 2008; Silberstein et al., 2011) and mice (Psotka et al., 2009; Jeong et al., 2018) injected with Stx have been used as valid experimental models of HUS to study the Stx-induced tubular pathophysiology in the renal cortex and medulla.

To date, no specific therapy is available to protect people from damage caused by STEC. Supportive care, such as intravenous volume expansion, fluid and electrolyte management, and peritoneal dialysis, is applied to reduce mortality in pediatric patients with HUS (Cody and Dixon, 2019; Balestracci et al., 2021). In this regard, our group has been investigating a possible strategy to prevent the cytotoxic action of Stx2 on human renal cells. Recently, we have demonstrated that the pre-incubation of HRTEC primary cultures with the drug eliglustat (EG), a selective and potent inhibitor of glucosylceramide synthase, which catalyzes the first step in glycosphingolipid biosynthesis (Supplementary Figure S1A), significantly reduced the Gb3 expression in the human renal tubular cells (Sánchez et al., 2021). Consequently, the treatments of HRTEC with pure EG totally prevented the reduction of cell viability, the induction of apoptosis and necrosis, and the inhibition of tubulogenesis caused by the exposure of cells to Stx2 (Sánchez et al., 2021).

Different inhibitors of glucosylceramide synthase have been identified and assayed for substrate inhibition therapy for the treatment of several glycosphingolipidoses, such as Fabry and Gaucher diseases (Shayman and Larsen, 2014). Currently, the drug eliglustat is used for oral substrate reduction therapy in adult patients with Gaucher disease to decrease the aberrant lysosomal accumulation of glycolipids such as Gb3 (Cox et al., 2015; Cox et al., 2017).

Based on our *in vitro* results with EG in HRTEC primary cultures, the present work aimed to investigate whether short-term oral treatment with EG can protect and prevent mortality and renal damage caused by Stx2 in lethal and sublethal experimental models of HUS in rats. For this purpose, juvenile male rats were intraperitoneally injected with either a lethal or a sublethal dose of Stx2, and different oral treatments with EG were assayed.

## 2 Materials and methods

### 2.1 Reagents

Purified Stx2a was purchased at Phoenix Lab, Tuft Medical Center, Boston, MA, USA, and was dissolved in sterile phosphate-buffered saline (PBS) solution (10 mmol/L sodium phosphate buffer and 0.9% NaCl, pH 7.4) before injection.

The drug eliglustat, also known as Cerdelga, was granted by the Sanofi Company. Each capsule contains 84 mg EG (as tartrate) and excipients (106 mg lactose as monohydrate, 45 mg cellulose, and others).

The Gb3 standard was purchased from Matreya (State College, PA, USA).

Antibodies: Rabbit anti-neutrophil gelatinase-associated lipocalin (NGAL) polyclonal antibody (pa5-88079; Invitrogen, Thermo Fisher Scientific, Argentina); rabbit IgG anti-aquaporin 1 (AQP1) polyclonal antibody (AQP11-A; Alpha Diagnostic, San Antonio, USA); and Alexa Fluor™ 555 (A-31572) goat-conjugated anti-rabbit IgG secondary antibody (A-31572; Invitrogen, Waltham, MA, USA).

### 2.2 Animal models and experimental design

Recently weaned Male Sprague–Dawley rats (∼100 g bw) were acquired from the animal facility at the School of Pharmacy and Biochemistry. Protocols for animal studies were approved by the Institutional Committee for the Care and Use of Laboratory Animals, Buenos Aires University (protocol no. 290/19-4081/04). The animals were housed under controlled conditions of a dark–light (12:12 h) cycle and temperature (22 °C–24 °C) and received water and food *ad libitum*.

To carry out the different treatments, rats were randomly divided into groups and placed in individual cages. To develop the lethal and sublethal experimental models of HUS, rats were intraperitoneally (ip) injected with 300 µL of a lethal (L_S_: 5 ng/g bw) or a sublethal (S_S_: 1 ng/g bw) dose of pure Stx2, respectively. Control rats were ip injected with the same volume of PBS. A time-course diagram of the experimental protocols carried out with lethal and sublethal doses of Stx2 in rats is shown in Supplementary Figures S1B,C.

For oral EG treatment, the capsule content was weighed, and 15 mg or 25 mg of EG was dissolved in 1 mL of distilled water. Daily food consumption was previously monitored to establish baseline intake and the intake during the treatments. A predetermined amount of food pellets was embedded with 1 mL of EG solution containing the full daily dose, adjusted according to the expected consumption in each group. The amount of food provided was reduced as needed to match decreased appetite while maintaining the full therapeutic dose. Daily weighing of residual food confirmed consumption of the EG-containing portion.

To evaluate the EG preventive effects caused by Stx2, the following treatments (Supplementary Figures S1B,C) were assayed:

1E-L_S_-2E: Rats were ip injected with the lethal dose of Stx2 and orally treated with EG (15 mg/d or 25 mg/d) for 3 days, starting from 1 day before toxin injection until 2 days post-injection (dpi).

2E-L_S_-2E: Rats were ip injected with the lethal dose of Stx2 and orally treated with EG (15 mg/d or 25 mg/d) for 4 days, starting from 2 days before toxin injection until 2 dpi.

1E-S_S_-2E: Rats were ip injected with the sublethal dose of Stx2 and orally treated with EG (25 mg/d) for 3 days, starting from 1 day before toxin injection until 2 dpi.

S_S_-3E: Rats were ip injected with the sublethal dose of Stx2 and orally treated with EG (25 mg/d) for 3 days, starting from the same day as the toxin injection until 3 dpi.

3E: Rats were ip injected with 300 µL PBS and orally treated with EG (25 mg/d) for 3 days, starting from 1 day before PBS injection until 2 dpi.

4E: Rats were ip injected with 300 µL PBS and orally treated with EG (15 mg/d or 25 mg/d) for 4 days, starting from 2 days before PBS injection until 2 dpi.

Some of the rats in each group were used for survival analysis, and they were monitored for food intake, water intake, and disease symptoms. Rats were weighed daily, and Δbody weight (Δbw) was calculated as bw at *x* day minus bw at 3 days before Stx2 or PBS ip injection.

To evaluate the renal function, the other rats were individually placed in metabolic cages at 2 days or 3 days post-Stx2 or PBS injection, for 24 h with water *ad libitum*. Urine was collected in the metabolic cages to measure the urinary flow. During this period, rats had no access to food to prevent contamination of urine with food particles and to allow accurate measurement of urinary biochemical parameters. All experimental groups were subjected to the same housing conditions. Subsequently, rats were euthanized using a carbon dioxide chamber.

### 2.3 Thin-layer chromatography (TLC) for Gb3 analysis

The expression of the Gb3 receptor was analyzed in renal samples of rats by TLC, as described previously (Sánchez et al., 2021). For this purpose, a group of rats was orally treated with EG (15 mg/d or 25 mg/d) for 1 day, 2 days, and 3 days. Control non-treated rats received the same amount of food without EG. After treatments, the kidneys were removed, and 100 mg of renal tissue was homogenized for 20 min in 0.8 mL of bi-distilled water, using an ULTRA-TURRAX (T25 basic, IKA, Labortechnik). Then, samples were syringed through 21 G and 27 G needles for 20 min, and finally, vortexed for another 20 min. Glycolipids were then extracted in chloroform:methanol:water (1:2:1 v/v) and submitted to alkaline hydrolysis in methanol and NaOH (1.0 N) for 16 h at 37 °C, following the protocol of Bligh and Dyer (1959), with some modifications described previously (Sánchez et al., 2021). Aliquots of glycolipid extracts were dissolved in chloroform–methanol (2:1) and separated by TLC in a mix of chloroform, methanol, and water (65:35:8). Samples were compared to a purified Gb3 standard (0.5–2.0 μg). The glycolipid bands were revealed after treating the TLC plate with a solution of orcinol. The densitometric analysis of Gb3 bands was performed by the ImageJ software (NIH).

### 2.4 Biochemical analysis, renal function, and water balance

Blood samples were obtained by cardiac puncture prior to euthanasia, without using an anticoagulant. Serum was obtained after blood centrifugation. Serum and urine creatinine and urea concentrations were measured using commercial kits (Wiener Lab, Argentina). To evaluate the glomerular filtration rate, the creatinine clearance (C_Creat_) was calculated as urine creatinine concentration × urinary flow/serum creatinine concentration.

Urinary protein levels were assessed using semiquantitative reagent strips (Combur 10 Test®, Roche), following the manufacturer’s instructions. Results were recorded using the manufacturer’s scoring scale as negative, 1+ (∼30 mg/dL), 2+ (∼100 mg/dL), or 3+ (∼500 mg/dL).

### 2.5 Tissue process for histopathology

Anesthetized rats were immediately fixed by intravascular perfusion with 10% formalin in PBS. The kidneys were removed and immersed in the same fixing solution. Tissue samples were then dehydrated and embedded in paraffin. Renal sections of 3 µm were deparaffinized, hydrated, and stained with periodic acid–Schiff (PAS). Renal sections were observed under an optical microscope (Nikon Eclipse 2000; NY, USA). For histopathological analysis, 10 to 15 images of cortical and medullary fields per kidney were captured using a digital camera (Nikon Coolpix 4300) under light microscopy from sections of three to six rats per group. Micrographs were processed using the ImageJ (NIH) analysis software. To evaluate the tubular damage, the percentage of tubules per field showing tubule dilation, cell nuclei loss, and brush border loss in proximal tubules was analyzed in cortical renal sections, as described in other reports (Zotta et al., 2008; Dennhardt et al., 2018). The percentage of tubule dilation and tubules with nuclei in the lumen was calculated in kidney medullary sections from the analysis of 10–15 fields per renal section, as described previously (Fischer Sigel et al., 2024). Histopathological evaluation was performed independently by two trained investigators, unaware of the experimental group.

### 2.6 Immunofluorescence

Histological sections were deparaffinized and hydrated, followed by antigen retrieval in 100 mmol/L citrate buffer, pH: 6.0 (98 °C, for 1 h), and permeabilized in 0.1% Triton X-100 in PBS (Fischer Sigel et al., 2024). Renal sections were blocked with 5% fetal bovine serum in PBS, followed by indirect immunofluorescence. For AQP1 and NGAL detection, renal sections were incubated with rabbit anti-AQP1 (1:100) or rabbit anti-NGAL (1:100) primary antibodies, respectively, followed by Alexa Fluor™ 555 goat-conjugated anti-rabbit IgG (1:500) secondary antibody. Fluorescence was observed with a fluorescence microscope. Ten images per renal section, from two or three rats per group, were captured with a digital camera Nikon E4300, and the integrated optical density (IOD) values were quantified using ImageJ (National Institutes of Health) software.

### 2.7 Statistical analysis

Results are reported as the mean ± standard error of the mean (SEM). One-way or two-way analysis of variance followed by the Tukey post-hoc test was used for comparison of three or more groups. The Kruskal–Wallis test for non-parametric data, followed by the Dunn post-hoc test, was used to calculate statistical significance for multiple comparisons. The Mann–Whitney test was used for non-parametric data for individual comparisons between two groups. A *p*-value <0.05 was considered significant. For TLC analysis, data are presented as the mean ± standard deviation (mean ± SD). The statistical analysis was performed using GraphPad Prism 6 software.

## 3 Results

### 3.1 Renal expression of Gb3 receptor in rats treated with EG

**FIGURE 1:**
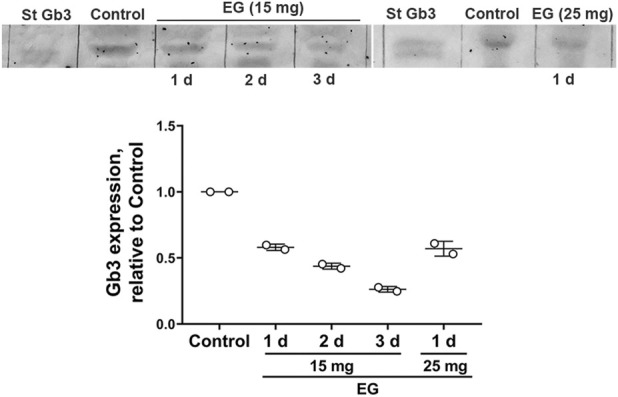
Eliglustat oral treatment reduces Gb3 renal expression in rats. Rats were orally treated with 15 mg EG/day for 1–3 days or 25 mg EG/day for 1 day. Control rats were not treated with EG Gb3 (1 µg) standard (St), and glycolipids extracted from rat renal tissues were separated by thin-layer chromatography followed by detection with orcinol. The graphic shows the respective densitometric analysis of Gb3 bands. Data are expressed as mean ± SD from two independent experiments.

Gb3 expression was evaluated by TLC in renal samples obtained from rats orally treated with EG (15 mg/d or 25 mg/d) and compared with renal samples of non-treated rats (Figure 1). The oral treatment with EG for 24 h was enough to decrease Gb3 renal expression to approximately 50% with respect to the control sample, without showing significant differences between the two EG doses (Figure 1). The treatment with EG for longer periods further decreased Gb3 expression in rat kidneys.

### 3.2 Survival analysis

**FIGURE 2:**
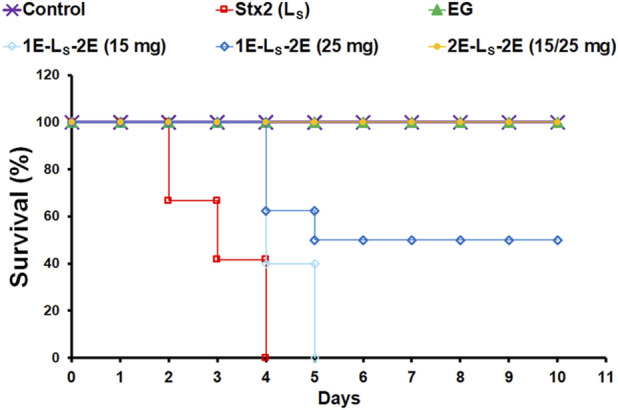
Rat survival. The graphic shows survival curves of rats intraperitoneally injected with a lethal (5 ng/g bw) dose of Stx2 (L_S_) or PBS (control). Some rats were orally treated with 15 mg or 25 mg EG/d, starting from 1 day (1E-L_S_-2E) or 2 days (2E-L_S_-2E) prior to 2 days post-Stx2 injection or eluent injection (EG), n = 6–10 rats per group.

Figure 2 shows the survival analysis of rats under the different treatments assayed, up to 10 dpi. All rats ip injected with the lethal dose of Stx2 died between 2 dpi and 4 dpi. These rats showed signs of piloerection, porphyrin in their eyes, reduction in activity, and finally lethargy. In the 2E-L_S_-2E group of rats, the oral treatments with EG with either of the two doses tested (15 mg/d or 25 mg/d) for 4 days, starting from 2 days before the Stx2 injection until 2 dpi, totally prevented rat mortality caused by the lethal dose of Stx2. Moreover, the treatment with 25 mg EG/d in the 1E-L_S_-2E group, starting from only 1 day before Stx2 injection until 2 dpi, prevented rat mortality by 50%, and the remaining rats died at 4 dpi to 5 dpi. However, all rats treated with 15 mg EG/d in the 1E-L_S_-2E group died at 4 dpi to 5 dpi.

All rats ip injected with the sublethal dose of Stx2 survived during the study and showed piloerection as the only visible symptom. Control non-treated rats as well as those orally treated with EG and injected with PBS (3E and 4E) or Stx2 sublethal dose (1E-S_S_-2E and S_S_-3E) also survived throughout the study.

### 3.3 Food intake, water intake, and body weight

**FIGURE 3:**
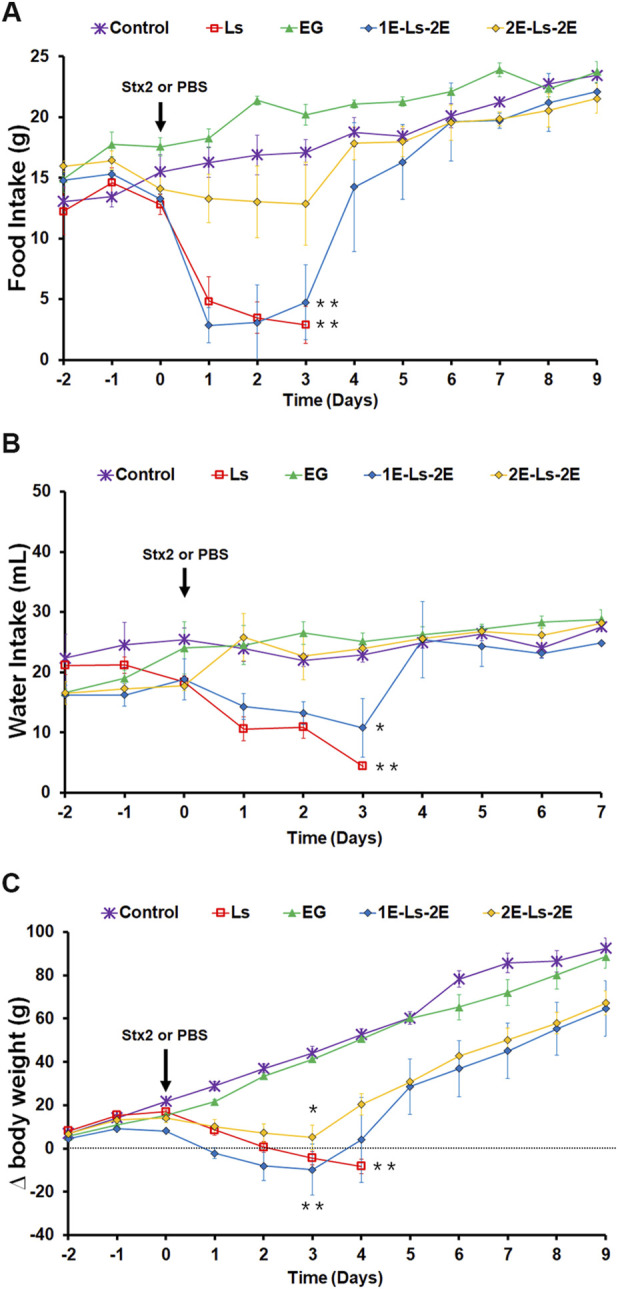
Food and water intake and body weight. Graphics show the time course of food **(A)** and water **(B)** intake and Δbody weight (Δbw) **(C)** per day from rats intraperitoneally injected with a lethal (5 ng/g bw) dose of Stx2 (L_S_) or eluent (control). Some rats were orally treated with 15 mg or 25 mg EG/d starting from 1 day (1E-L_S_-2E) or 2 days (2E-L_S_-2E) prior to 2 days post-Stx2 injection or eluent injection (EG). Each point of the curves represents the mean ± SEM, n = 6 rats. ANOVA for repeated measures followed by Tukey post-hoc test indicates significant differences: *p < 0.05 and **p < 0.01 vs. control rats, and vs. rats treated with EG and injected with eluent.

Figures 3A–C show curves of food intake, water intake, and Δbody weight, respectively, of the different groups of rats over time. Control rats and rats treated with either dose of EG gradually increased food intake, maintained water intake, and significantly increased their body weight throughout the days.

Rats ip injected with the lethal (L_S_) dose of Stx2 significantly decreased the food and water intake from the first dpi until their death, and consequently, significantly reduced their body weight (Figures 3A–C). The 2E-L_S_-2E rats, orally treated with either 15 mg or 25 mg of EG per day, slightly reduced their food intake, maintained their water intake, and significantly decreased their body weight between the first and third day post-Stx2 injection. At 4 dpi, this group of rats recovered their appetite and thirst and began to regain body weight. However, the 1E-L_S_-2E rats, pre-treated with 25 mg EG from only 1 day prior to the Stx2 lethal dose inoculation, significantly reduced water and food intake and body weight, similar to the L_S_ rats. Those 1E-L_S_-2E rats that survived returned to pre-test parameter levels by the 4th dpi.

Rats injected with the sublethal dose of Stx2, treated or not with EG, showed similar values of food intake and body weight throughout the days, as observed in control rats (data not shown). However, S_S_ rats transiently increased water intake with a peak at 4 dpi compared with S_S_ rats at 3 dpi (S_S_, 4 dpi: 28 ± 1.9 mL/d 100 g bw vs. 3 dpi: 18 ± 2.4 mL/d 100 g bw, n = 6). None of the EG treatments in rats injected with the Stx2 sublethal dose caused significant modifications in these parameters, compared with control rats (data not shown).

### 3.4 Renal functional parameters

**FIGURE 4:**
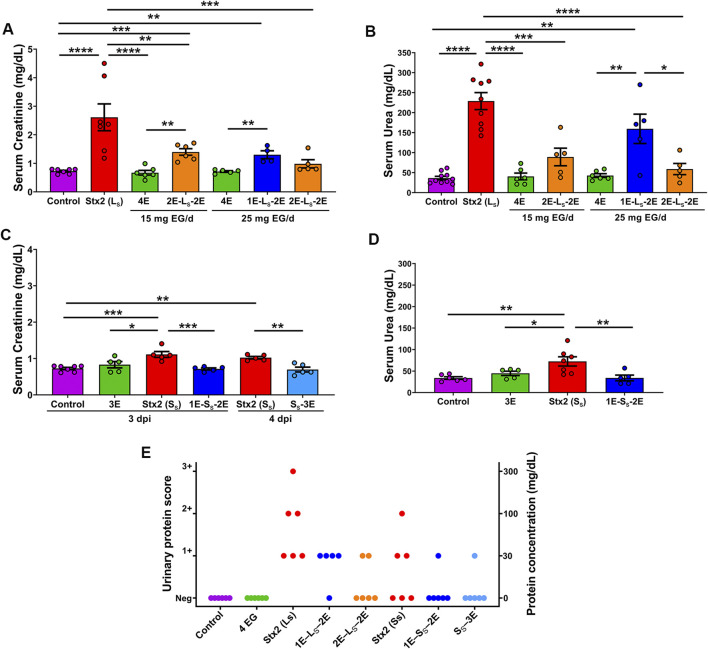
Serum creatinine and urea levels. Graphics show serum creatinine **(A,C)** and serum urea **(B,D)** levels from rats ip injected with a lethal **(A,B)** or a sublethal **(C,D)** dose of Stx2 or PBS, and orally treated or not with 15 mg or 25 mg EG/d, at 3 dpi or 4 dpi. Data are expressed as mean ± SEM, n = 5–9 rats per group. One-way ANOVA followed by the Tukey post-hoc test indicates significant differences: *p < 0.05, **p < 0.01, ***p < 0.001, and ****p < 0.0001. The graphic in panel **(E)** shows urinary proteinuria levels of the different groups of rats. Each dot represents the proteinuria value for each rat (n = 6 rats per group). Results were recorded using the manufacturer’s scoring scale as negative, 1+ (∼30 mg/dL), 2+ (∼100 mg/dL), or 3+ (∼500 mg/dL).

To evaluate the protective effect of EG on renal function in rats injected with lethal and sublethal doses of Stx2, serum creatinine and urea levels (Figure 4) and C_Creat_ (Table 1) were measured at 3 dpi or 4 dpi, depending on the extent of post-injection treatment.

**TABLE 1:** Renal parameters. Creatinine clearance and urinary flow were evaluated in rats orally treated or not with 15 mg or 25 mg EG/day, at 3 days and 4 days post ip injection with lethal or sublethal doses of Stx2 or eluent. One-way ANOVA followed by the Tukey post-hoc test indicates significant differences (n = 5–6 rats per group).

Treatment	Creatinine clearance (mL/min.100 g bw)	Urinary flow (mL/d.100 g bw)
Control	0.49 ± 0.111	20.1 ± 3.42
Stx2 lethal dose	0.09 ± 0.024**^##^	12.1 ± 2.13*^#^
EG 15 mg	0.46 ± 0.130	23.1 ± 5.64
2E-L_S_-2E (15 mg)	0.19 ± 0.040*^#&^	17.7 ± 2.86
EG 25 mg	0.47 ± 0.124	21.7 ± 4.62
1E-L_s_-2E (25 mg)	0.23 ± 0.062*^&^	25.4 ± 3.90
2E-L_S_-2E (25 mg)	0.39 ± 0.089^&&^	23.7 ± 4.68
Stx2 sublethal dose (3 d)	0.19 ± 0.061*^#&&^	20.0 ± 0.95
1E-S_S_-2E	0.32 ± 0.036	22.1 ± 1.64
Stx2 sublethal dose (4 d)	0.24 ± 0.043*	29.1 ± 2.60*^&&^
S_S_-3E	0.32 ± 0.071	22.0 ± 5.00

*p < 0.05 and **p <0.01 vs control rats; #*p* < 0.05 and ##*p* < 0.01 vs rats orally treated with EG and injected with eluent; &*p* < 0.05 and &&p < 0.001 vs rats injected with Stx2 lethal or sublethal dose.

Rats orally treated with 15 mg or 25 mg EG/d and injected with PBS showed normal values of serum creatinine, urea, and C_Creat_, as shown in control rats. The lethal dose of Stx2 caused a significant increase in serum creatinine and urea concentrations (Figures 4A,B) and a significant decrease in C_Creat_ (Table 1) compared to the control and EG-treated rats. The oral treatments with 15 mg EG/d in 2E-L_S_-2E rats and with 25 mg EG/d in 1E-L_S_-2E rats partially but significantly prevented the increase of serum creatinine concentration and the decrease of C_Creat_ compared with L_S_ rats. The treatment with 15 mg EG/d in 2E-L_S_-2E rats also prevented the serum urea increase. However, the treatment with 25 mg EG/d did not prevent the increase of serum urea in 1E-L_S_-2E rats, demonstrating that the pretreatment period is important to prevent the effects of the toxin. The oral treatment with 25 mg EG/d in 2E-L_S_-2E rats totally prevented the alterations of renal functional parameters caused by the Stx2 lethal dose, showing no significant differences compared with the Control and 4E groups at 3 dpi (Figures 4A,B; Table 1).

In S_S_ rats, the sublethal dose of Stx2 induced a slight but significant increase in serum creatinine levels (Figure 4C) and a decrease in C_Creat_ (Table 1) compared with control and 3E rats at 3 dpi and 4 dpi. At 3 dpi, the Stx2 sublethal dose caused a mild transient and significant increase of serum urea values in the group of S_S_ rats (Figure 4D), while at 4 dpi, serum urea recovered to normal levels without showing significant differences compared with control and 3E rats (data not shown). The alteration of renal parameters was significantly lower in S_S_ than in L_S_ rats (Figure 4; Table 1).

To evaluate the effect of EG in rats inoculated with the Stx2 sublethal dose, the animals were orally treated with 25 mg EG/d. The treatment with EG from only 1 day prior to the Stx2 sublethal dose injection totally prevented the increase of serum creatinine and urea in 1E-S_S_-3E rats (Figures 4C,D). In S_S_-3E rats, the oral treatment with 25 mg EG/d, starting at the same time as the Stx2 sublethal dose injection, also prevented the serum creatinine increase (Figure 4C). Even though both 1E-S_S_-3E and S_S_-3E rats showed a tendency to increase the C_Creat_ compared to S_S_ rats, the increase was not significant (Table 1).

Proteinuria, particularly albuminuria, was detected in the urine of rats from the different experimental groups, using semiquantitative reagent strips. Control and EG-treated rats showed no detectable proteinuria, with all animals presenting negative scores (Figure 4E). In contrast, rats injected with the Stx2 lethal dose exhibited proteinuria, with all animals showing positive scores, including moderate levels (1+) and higher scores (≥2+). As expected, rats injected with the Stx2 sublethal dose showed lower or undetectable proteinuria levels. EG treatment reduced both the frequency and severity of proteinuria in lethal and sublethal Stx2 rat models, with most treated animals presenting negative or low (1+) proteinuria scores (Figure 4E).

Urinary flow was also analyzed at 3 dpi or 4 dpi (Table 1). Oral treatments with any of the EG doses did not significantly modify animal diuresis compared to control rats. The lethal dose of Stx2 significantly decreased the urinary flow at 3 dpi compared with the values of control rats. The oral treatments with EG prevented the diuresis alteration in 1E-L_S_-2E and 2E-L_S_-2E rats, at 3 dpi (Table 1).

On the other hand, the Stx2 sublethal dose caused a significant increase in urinary flow in S_S_ rats at 4 dpi, compared with the control and EG-treated rats (Table 1). These results are consistent with the increased water intake shown in the same group of animals, as described above. The oral treatments with EG from the same day (S_S_-3E) of Stx2 sublethal dose injection prevented the urinary flow alteration.

### 3.5 Histomorphology

**FIGURE 5:**
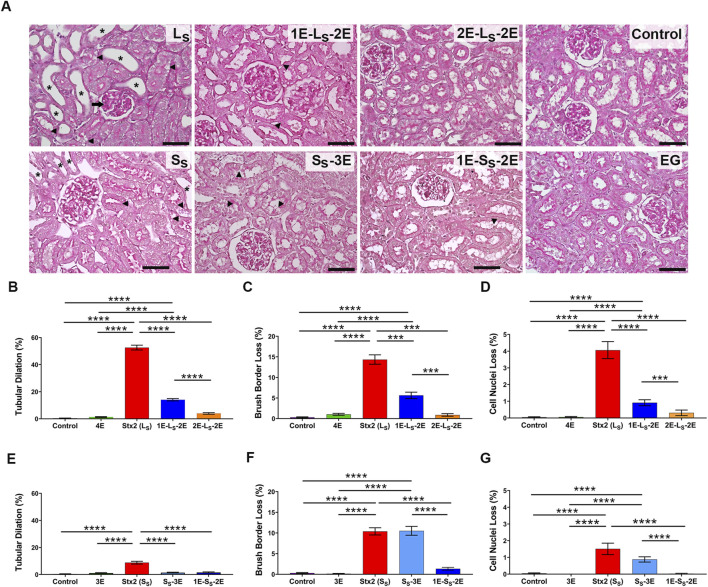
Renal histomorphology in cortical sections. **(A)** Representative microphotographs show renal cortical sections stained with periodic acid–Schiff, from rats ip injected with lethal (L_S_: 5 ng/g bw) or sublethal (S_S_: 1 ng/g bw) doses of Stx2 or eluent (control). Some rats injected with a lethal dose of Stx2 were orally treated with 25 mg EG/d starting from 1 day (1E-L_S_-2E) or 2 days (2E-L_S_-2E) pre-injection to 2 days post-Stx2 injection or eluent injection (EG). Some rats injected with the Stx2 sublethal dose were orally treated with 25 mg EG/d starting from 1 day (1E-L_S_-2E) pre-injection to 2 days post-injection, or on the same day (S_S_-3E) of injection until 3 days post-injection. Asterisks indicate tubular dilation; arrowheads indicate epithelial brush border and loss of nuclei. The arrow in the glomerulus indicates mesangial proliferation. Scale bars = 50 μm. Graphics in **(B–D)** show the percentage of tubular dilation, brush border loss, and nuclei loss, respectively, in rats injected with a Stx2 lethal dose. Graphics in **(E–G)** show the percentage of tubular dilation, brush border loss, and nuclei loss, respectively, in rats injected with Stx2 sublethal dose. Data are expressed as the mean ± SEM of 10–15 fields per section of three rats. Kruskal–Wallis test followed by the Dunn post-hoc test indicates significant differences: **p < 0.01, ***p < 0.001, and ****p < 0.0001.

Representative micrographs in Figure 5A show cortical renal sections stained with PAS obtained from the different groups of rats at 3 dpi. The percentage of tubules per field with tubular dilation, cell nuclei loss, and loss of brush border in proximal tubules was quantified from cortical renal sections of the different groups of rats (Figures 5B–G). Conserved renal histomorphology was observed in the kidneys of control rats and rats treated with 25 mg EG/d for 3 days or 4 days.

The Stx2 lethal dose caused extensive renal damage, demonstrated by the increased percentage of dilated tubules with epithelial flattening (Figure 5B) and proximal tubules showing brush border and cell nuclei loss (Figures 5C,D), consistent with the tubular necrosis observed in L_S_ rats compared with control and EG-treated rats. In addition, mesangial proliferation was observed in the glomeruli of some L_S_ rats (Figure 5A).

In 1E-L_S_-2E rats, the oral treatment with 25 mg EG/d from 1 day prior to the Stx2 injection partially and significantly prevented the renal histological damage, demonstrated by the significantly lower percentage of tubules with epithelial necrosis and epithelial flattening compared with the kidneys of L_S_ rats. In 2E-L_S_-2E rats, in contrast, the oral treatment with 25 mg EG/d from 2 days prior to the Stx2 injection almost totally prevented the renal histological damage caused by the Stx2 lethal dose. Renal sections of these rats showed conserved histomorphology similar to that of the control and EG-treated rats (Figure 5A).

Cortical renal sections of S_S_ rats inoculated with the Stx2 sublethal dose showed mild to moderate tubular injury, with a lower percentage of tubular dilation (Figure 5E), focal tubular necrosis, including tubular nuclei and brush border loss (Figures 5F,G) in proximal tubules, than in L_S_ kidneys. Glomeruli histomorphology was not significantly altered in S_S_ rats. The oral treatment with 25 mg EG/d in 1E-S_S_-3E totally prevented the renal histological injury produced by the sublethal dose of Stx2 at 3 dpi and showed a conserved renal histomorphology. However, EG treatment from the same day of Stx2 inoculation prevented tubule dilation but did not prevent brush border and cell nuclei loss in S_S_-3E rats (Figures 5E–G).

**FIGURE 6:**
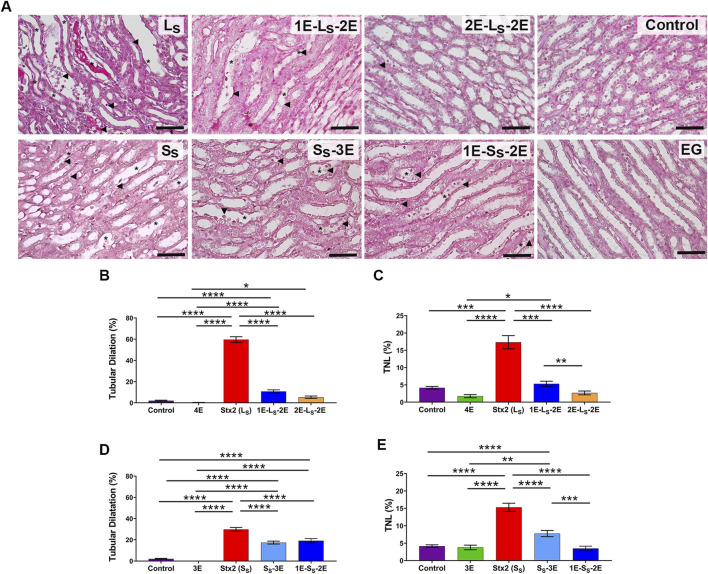
Renal histomorphology in medullary sections. **(A)** Representative microphotographs show renal medullary sections stained with periodic acid–Schiff from rats intraperitoneally injected with lethal (L_S_: 5 ng/g bw) or sublethal (S_S_: 1 ng/g bw) doses of Stx2 or eluent (control). Some rats injected with a lethal dose of Stx2 were orally treated with 25 mg EG/d starting from 1 (1E-L_S_-2E) or 2 (2E-L_S_-2E) days pre-injection to 2 days post-Stx2 injection or eluent injection (EG). Some rats injected with the Stx2 sublethal dose were orally treated with 25 mg EG/d starting from 1 day (1E-L_S_-2E) pre-injection to 2 days post-injection, or on the same day (S_S_-3E) of injection until 3 days post-injection. Asterisks indicate tubular dilation; arrowheads indicate tubules with a loss of nuclei in the lumen (TNL). Scale bars = 50 μm. Graphics in **(B,C)** show the percentage of tubular dilation and TNL, respectively, in rats injected with a lethal dose of Stx2. Graphics in **(D,E)** show the percentage of tubular dilation and TNL, respectively, in rats injected with Stx2 sublethal dose. Data are expressed as the mean ± SEM of 10–15 fields per section of three rats. Kruskal–Wallis test followed by the Dunn post-hoc test indicates significant differences: *p < 0.05, **p < 0.01, ***p < 0.001, and ****p < 0.0001.

Figure 6A shows representative micrographs of medullary renal sections stained with PAS obtained from the different groups of rats. Graphics with the corresponding quantification of tubular dilation and percentage of tubules showing nuclei loss in the lumen are shown in Figures 6B,C, respectively.

The Stx2 lethal dose significantly increased the percentage of tubular dilation and tubules with nuclei in the lumen in medullary sections of L_S_ rats, compared with the control and 4E rats, which showed a conserved histology. The Stx2 sublethal dose caused significantly less tubular damage than the lethal dose. The treatment with 25 mg EG/d, in both 1E-L_S_-2E and 2E-L_S_-2E rats, significantly prevented the damage caused by Stx2 lethal dose, depending on the amount of pretreatment time (Figures 6B,C). The oral treatment with EG significantly decreased the tubular alterations caused by the Stx2 sublethal dose in 1E-S_S_-2E and S_S_-3E rats (Figures 6D,E).

### 3.6 NGAL and AQP1 tubular expression

**FIGURE 7:**
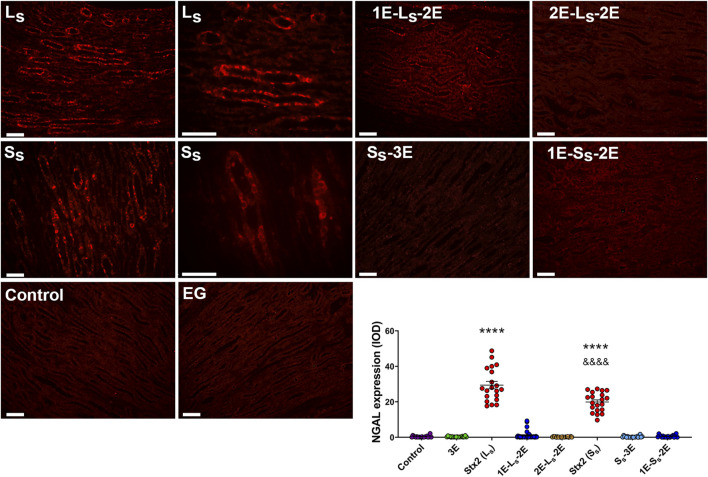
NGAL immunofluorescence in renal sections. NGAL-positive expression is shown in panoramic (left) and amplified (right) representative microphotographs of renal medullary tubules from rats injected with lethal (L_S_) and sublethal (S_S_) doses of Stx2, at 3 days post-Stx2 injection. Scale bar = 50 μm. The graphic represents IOD values of NGAL-positive staining. Data are expressed as the mean ± SEM of 10 fields per section of two rats. The Mann–Whitney test was used to indicate significant differences: ****p < 0.0001 for Stx2 (L_S_) and Stx2 (S_S_) vs. the remaining treatments; ^&&&&^ *p* < 0.0001 for Stx2 (S_S_) vs. Stx2 (L_S_).

The immunofluorescence for NGAL corroborated the results obtained from histological observations in renal sections. Representative micrographs in Figure 7 show positive staining for NGAL, a marker of acute tubular injury, on tubular epithelia located in the renal medulla of rats injected with lethal and sublethal doses of Stx2, at 3 dpi. A detail of NGAL labeling is shown in higher magnification kidney photographs of L_S_ and S_S_ rats (Figure 7). The graphic in Figure 7 shows that significantly higher IOD values were obtained from NGAL-positive tubules in renal sections of L_S_ rats than in those of S_S_ rats. All oral treatments with EG almost completely prevented NGAL expression in renal sections of rats injected with both the lethal and sublethal doses of the toxin. Control and EG-treated rats did not show NGAL staining in tubules.

**FIGURE 8:**
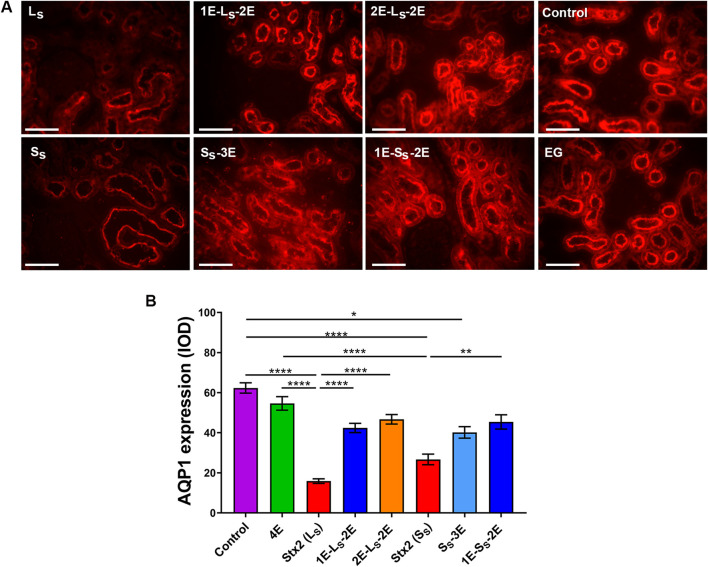
AQP1 immunofluorescence in renal sections. **(A)** AQP1-positive expression is shown in representative microphotographs of renal cortical tubules from control rats and rats injected with lethal (L_S_) and sublethal (S_S_) doses of Stx2, with or without eliglustat (EG) treatment, at 3 days post-Stx2 injection. Scale bar = 50 μm. **(B)** The graph represents the IOD values of AQP1-positive staining. Data are expressed as the mean ± SEM of 10 fields per section from two rats per group. The Kruskal–Wallis test was used to indicate significant differences: *p < 0.05, ****p < 0.0001.

Indirect immunofluorescence was performed to evaluate AQP1 expression as a marker of renal proximal tubule integrity in renal sections of rats with different treatments. As shown in representative micrographs of Figure 8A, AQP1 is localized in both apical and basolateral membranes of renal proximal tubule epithelial cells. The Stx2 lethal dose caused a significant reduction of AQP1 expression, measured as IOD, in the renal proximal tubules compared to the kidneys of control and EG-treated rats (Figure 8B). In addition, discontinuous areas in AQP1 expression at the luminal membrane of proximal epithelial cells are shown, corroborating the brush border damage observed in the kidneys of L_S_ rats compared with those of control and EG-treated rats (Figure 8A). In the kidneys of S_S_ rats, a significantly smaller reduction of AQP1 expression was observed than in L_S_ rats. Oral treatments with EG prevented the alterations in AQP1 expression caused by Stx2 in the proximal tubule, according to the Stx2 dose and the EG-pretreatment times. These results are consistent with histological observations.

## 4 Discussion

In the present study, we have evaluated the efficacy of short transient oral treatments with the drug EG on the prevention of renal injury caused by lethal and sublethal doses of Stx2 ip injected in juvenile male rats. The rat HUS models used for this work developed some of the renal pathophysiological features of human HUS post-STEC infection. Different studies have demonstrated the relevance of severe tubular damage caused by Stx, detected in human renal histological sections (Uchida et al., 1999; Chaisri et al., 2001; Porubsky et al., 2014) and by the increase in specific markers of tubular damage, such as NGAL, in the urine of patients with HUS (Trachtman et al., 2006). Both L_S_ and S_S_ rats recapitulated mainly the renal tubular damage previously reported in patients, making them an appropriate experimental tool to evaluate the effect of EG in preventing Stx2-induced renal pathogenesis.

The EG compound is a small ceramide analog molecule (Supplementary Figure S1A), which acts as an inhibitor of glucosylceramide synthase, a Golgi enzyme that catalyzes the formation of glucosylceramide from ceramide and uridine diphosphate-glucose and is the first step in the formation of glucocerebroside-based glycosphingolipids, including Gb3 (Shayman, 2010). In the present work, the lethal dose of Stx2 reduced food intake and therefore caused body weight loss. The oral treatments with EG allowed a rapid recovery of food intake and body weight in rats injected with the Stx2 lethal dose. Rats were orally treated with the capsule content of eliglustat tartrate, instead of the pure drug used previously for *in vitro* studies performed in cell culture, in order to promote its better absorption in the digestive tract of animals. In addition, the treatment with EG alone did not alter either the food intake or the body weight of the rats. Preclinical pharmacological studies in normal experimental animals demonstrated that EG tartrate contributed to a more effective absorption of EG in the digestive tract and consequently, to a better oral bioavailability of the drug. Moreover, it has a good pharmacokinetic and pharmacodynamic profile for use in humans and contains a natural ceramide structure, which could represent a significant advantage in terms of safety and clinical efficacy (McEachern et al., 2007; Shayman, 2010).

Short-term oral treatment with both EG doses was effective in reducing Gb3 expression in the kidney of rats. Partial significant reduction of renal Gb3 expression was sufficient to prevent rat mortality as well as renal injury caused by lethal and sublethal doses of Stx2 in rats. These results are consistent with those previously obtained in primary cultures of HRTEC, isolated from pediatric kidneys, in which the incubation with EG (500 nM), with a pretreatment of only 6 h, partially reduced Gb3 expression, and consequently, completely prevented Stx2 cytotoxic effects (Sánchez et al., 2021). In addition, another work conducted in primary human glomerular microvascular endothelial cells reported that the incubation with EG partially decreased cellular Gb3, glucosylceramide, and lactosylceramide levels, compared to control cells, which significantly decreased Stx binding to the cell surface and prevented the inhibition of protein synthesis caused by Stx2a (Feitz et al., 2021). Furthermore, in patients with Gaucher disease, EG acts by partially inhibiting the *de novo* biosynthesis of β-glucosylceramide, thereby rebalancing the formation rate of the primary substrate of the deficient enzyme with its impaired degradation (Cox et al., 2017).

The treatment with both 15 mg and 25 mg EG for only 1 day significantly reduced renal Gb3 expression without showing significant differences between doses. However, the degree of protection from Stx2 action depended on the daily dose and pretreatment duration. In fact, the oral treatments with 25 mg EG/d were significantly more effective in preventing the effects of Stx2 in rats than were the treatments with 15 mg EG/d. It is possible that the sensitivity of the TLC methodology was not sufficient to detect significant differences between the effects of both EG doses on Gb3 reduction. Moreover, EG treatment, according to the dose, could have modified the distribution of Gb3 in the cell membrane surface. The distribution of Gb3 in plasma membrane fractions and its arrangements in detergent-resistant membranes or lipid rafts have been reported, which could facilitate the Gb3 binding to Stx, affecting cell sensitivity to the toxin (Legros et al., 2017; Detzner et al., 2021).

The treatment with either EG dose (15 mg/d or 25 mg/d), from 2 days prior to 2 days post-Stx2 injection, prevented animal mortality by 100%. The EG pretreatment from 1 day prior to Stx2 inoculation partially prevented rat mortality by 50%, but only with the dose of 25 mg of EG/d. These results demonstrate that the pretreatment period is critical to the drug’s effectiveness. Moreover, the oral treatment with EG in rats injected with the Stx2 lethal dose was more effective than the compound C-9, another glucosylceramide synthase inhibitor, as assayed in a previous work conducted in rats that required 48-h pretreatment to achieve only 50% rat survival (Silberstein et al., 2011).

In the present work, rats injected with the Stx2 lethal dose showed increased serum creatinine and urea levels, reduction of urinary flow, and a notable reduction of the glomerular filtration rate at 3 dpi, which was consistent with the histopathological alterations observed in renal glomeruli. Stx2 caused significantly greater and more widespread histological damage at the tubular level, affecting both the renal cortex and medulla. Consistent with the creatinine levels and clearance, semiquantitative analysis revealed the presence of proteinuria in Stx2-injected rats, mainly in the lethal model, while EG treatments markedly reduced both the frequency and severity of proteinuria. Results in L_S_ rats are comparable to those previously described in rats injected with a lethal dose of bacterial supernatant expressing Stx2, in which significant microalbuminuria and downregulation of nephrin and podocalyxin expression in podocytes were observed at 48 h post-injection, potentially explaining the reduction of the glomerular filtration rate (Zotta et al., 2008; Ochoa et al., 2012). Other studies conducted with rats and mice injected with pure Stx2a showed significant tubular injury without the glomerular thrombotic microangiopathy characteristic of human HUS. These results have been attributed to the lack of Gb3 detection in glomerular endothelial cells in these animal species (Psotka et al., 2009; Obrig and Karpman, 2012; Porubsky et al., 2014). The possibility that a low Gb3 expression in rat glomerular endothelium and podocytes confers sensitivity to higher doses of Stx2 in rats has not been ruled out. In this regard, the same EG treatments that reduced renal Gb3 levels and protected the rats from mortality also prevented the AKI caused by Stx2 lethal dose, including glomerular alterations. Differences among animal models may also be due to the dose of Stx administered and bacterial lipopolysaccharide contamination (Clayton et al., 2005; Landoni et al., 2022).

In S_S_ rats, a shorter pretreatment with EG (25 mg/d) was required to significantly prevent kidney injury, compared with L_S_ rats, possibly because the renal injury in these animals was significantly lower than in rats injected with a lethal dose of Stx2. It is noteworthy that in S_S_-3E rats, EG also partially but significantly prevented the Stx2-kidney damage despite the absence of an EG-pretreatment period. However, EG prevented tubule dilation but did not prevent brush border and cell nuclei loss in S_S_-3E rats. It is possible that EG intake from the same day of Stx2 injection did not protect against direct primary damage from Stx2 upon contact with renal tubular epithelial cells, causing loss of brush border and cell nuclei. Renal tubular dilation can be a consequence of epithelial injury, which leads to cell flattening and loss of brush borders, creating a wider lumen. Therefore, EG treatment without a pretreatment period could have prevented subsequent dilation following direct injury. S_S_ rats showed a mild AKI with tubular epithelial histological damage, while the glomeruli maintained a normal appearance. Moreover, S_S_ rats showed an increased urinary flow at 4 dpi. These results were consistent with those previously found in adult female rats ip injected with a sublethal dose of Stx2 (Fischer Sigel et al., 2024) and in other experimental models of sublethal HUS in rats and mice (Psotka et al., 2009; Sugatani et al., 2002; Dennhardt et al., 2018). In those studies, polyuria was associated with reduced water reabsorption due to the reduced expression of aquaporin 2 in the tubular collecting duct principal epithelial cells caused by Stx.

The decreased and increased urinary flow found in rats injected with Stx2 lethal and sublethal doses, respectively, coincided with changes in water intake. The treatment with EG did not produce significant differences in the urinary flow, indicating that this drug does not alter the mechanism of urine formation. These findings coincide with those observed in another study in which rats that received an oral dose of 400 mg EG/kg did not present alterations in urinary flow or renal sodium excretion (Shayman, 2010).

Both lethal and sublethal Stx2 doses induced NGAL expression mainly in medullary renal tubules, consistent with the level of renal tubular histological lesion observed in L_S_ and S_S_ rats. NGAL is a biomarker of AKI and is upregulated and released after tubular damage and in response to inflammation (Romejko et al., 2023). In the kidney, NGAL is synthesized in the distal part of the nephron, especially in the ascending Henle’s loop and the collecting duct, which is consistent with our observations. Moreover, it is known that during HUS disease, pro-inflammatory cytokines increase and contribute to the inflammatory response (Clayton et al., 2005; Exeni et al., 2018). In our work, EG treatments almost totally prevented the renal tubular expression of NGAL, which could demonstrate the prevention of inflammatory response and tubular injury in the rat kidney.

It is known that Stx2 may act not only in the kidneys but also in other organs such as the intestine and nervous system (Repetto, 2005; Khalid and Andreoli, 2019), which may also have affected the health and mortality of the rats (Silberstein et al., 2011; Arenas-Mosquera et al., 2022). Future studies will be necessary to evaluate whether EG can prevent the effects of Stx2 on other organs in the same groups of rats.

The results of AQP1 expression corroborated the histological observations of brush border damage. In a previous work, we showed that Stx2 significantly reduced water reabsorption across human renal tubular epithelial cell monolayers without altering the paracellular water pathway (Silberstein et al., 2008). A more recent report by Gomez et al. (2025) showed that exposing human endothelial cell primary cultures to Stx2 significantly decreased AQP1 expression. Therefore, AQP1 may be considered a marker of renal proximal tubule integrity. Moreover, in this study, proteinuria caused by Stx2 could be due not only to glomerular alteration but also to proximal tubule damage. In the present work, we have assayed the drug, EG, in lethal and sublethal rat models of HUS in which Stx2 caused different levels of AKI, partially resembling human kidney injury, representing both patients who die due to post-diarrheal HUS and those who survive the disease with long-term sequelae (Repetto, 2005; Cobeñas et al., 2007). Oral treatment with EG was effective in attenuating kidney injury at the histological level and in preserving kidney function in rats intraperitoneally injected with lethal and sublethal doses of Stx2. In addition, EG treatments did not alter any of the parameters studied in rats. The results of these studies show the importance of both the oral dose per day and the time of pretreatment, depending on the Stx2-injected dose. Treatment with glucosylceramide synthase inhibitors, such as EG, could represent a potential alternative for AKI prevention or reduction of renal risk factors that can lead to chronic and end-stage kidney injury. Regarding this, recent pharmacokinetic modeling studies have proposed pediatric dosing regimens for EG in STEC-HUS, evidencing the translational potential of this therapeutic approach (Wolthuis et al., 2025).

HUS typically develops 3–7 days after the onset of bloody diarrhea (Tarr et al., 2005; Joseph et al., 2020). This prodromal period, combined with early detection of STEC infection, provides a critical window for intervention before the establishment of severe AKI. Therefore, EG could be administered for a few days within this period before kidney damage occurs in patients infected with STEC to protect the kidneys from Stx2 effects. It is important to clarify that in the animal models studied in the present work, the toxin was administered intraperitoneally, bypassing the intestinal phase of bacterial infection with STEC. Therefore, this model lacks the window period prior to the development of HUS in STEC-infected patients. However, this approach is clinically meaningful, based on the short-term preventive effect of the oral treatment with EG in both rat HUS models.

### 4.1 Conclusion

Taking into account that there is no specific treatment for HUS caused by STEC, and that Stx activity requires binding to the Gb3 receptor on the surface of target cells, our group has studied a glucosylceramide synthase inhibitor to indirectly reduce Gb3 expression and therefore prevent kidney injury caused by Stx2 in rats. In this work, oral EG treatment was shown to effectively prevent the cytotoxic effects of Stx2 in experimental models of lethal and sublethal HUS in rats. The oral treatment with EG prevented mortality in rats exposed to lethal doses of Stx2, as well as histological and functional kidney injury induced by lethal and sublethal doses of the toxin. EG tartrate therapy has been tested for prolonged periods of several years in patients with Gaucher disease (Cox et al., 2017). This may represent an advantage in the case of a possible implementation of EG for substrate reduction therapy, as a brief and transient treatment, to prevent the action of Stx and the development of AKI in patients with post-diarrhea HUS caused by STEC. This study provides the first *in vivo* evidence that the eliglustat prevents the action of Shiga toxin 2 and supports the potential translational use of this drug as a preventive or early-intervention strategy for diarrhea-associated hemolytic uremic syndrome.

## Data Availability

The original contributions presented in the study are included in the article/Supplementary Material; further inquiries can be directed to the corresponding author.
