# Supplementary figures and images for: Eliglustat prevents acute kidney injury caused by Shiga toxin 2 in lethal and sublethal rat models of hemolytic uremic syndrome
Source: Front Pharmacol. 2026 Feb 19;17:1736204. doi: 10.3389/fphar.2026.1736204 (PMC12960549; doi:10.3389/fphar.2026.1736204)

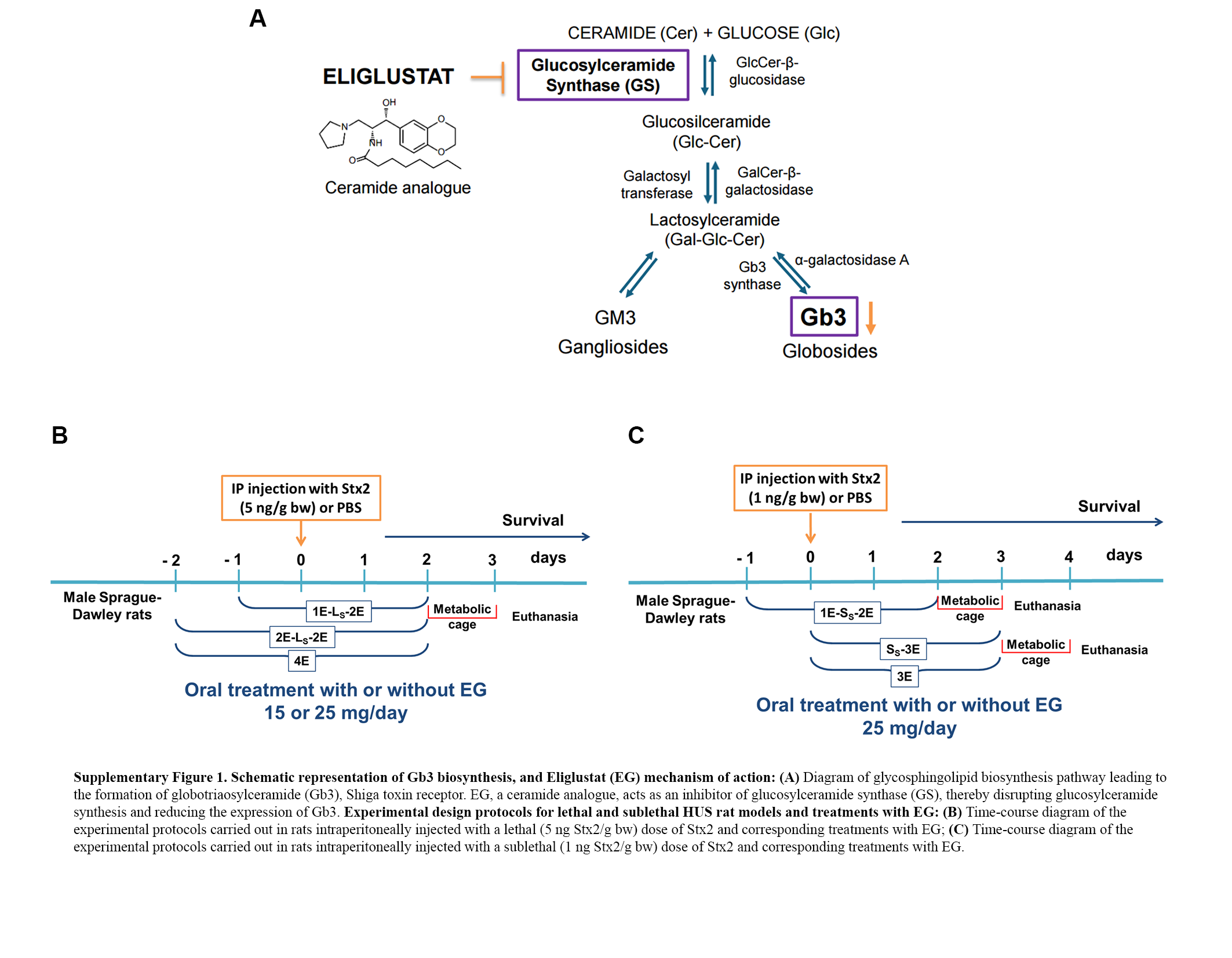

Supplement: Supplementary file 1 [file Image1.tif]
